# Perceptions of preceptorship in clinical practice after completion of a continuous professional development course- a qualitative study Part II

**DOI:** 10.1186/s12912-015-0092-8

**Published:** 2015-08-01

**Authors:** Elisabeth Carlson, Mariette Bengtsson

**Affiliations:** Department of Care Science, Faculty of Health and Society, Malmö University, Malmö, Sweden

**Keywords:** Advanced level, Clinical practice, Evaluation, Preceptor, Professional development

## Abstract

**Background:**

For health care professionals, clinical practice is a vital part of education, and in several countries, teaching is a regulated part of the role of nurses and health care staff. The added responsibility of taking on the teaching of students during clinical practice; thus, balancing clinical and educational demands, might lead to feelings of stress and burnout. Being a skilled and experienced professional is not automatically linked to being a skilled educator as teaching of a subject is a completely different story. Preceptors who participate in educational initiatives are better prepared to address challenges and are more satisfied with the preceptor role. The aim of the current study was to evaluate preceptors’ experiences of preceptorship in clinical practice after completion of a credit bearing continuous professional development course on advanced level.

**Methods:**

This was a small-scale interpretative qualitative study drawing data from focus group interviews and written accounts from reflective journals. Data were analysed through the process of naturalistic inquiry.

**Results:**

Our findings show that the participants, who took part in and completed the CPD course, had developed skills and competences they believed to be necessary to drive pedagogical development at their respective workplaces. This is illustrated by the main category Leading educational development and explained by four sub-categories: 1/ increased ability to give collegial support; 2/ increased trust in one’s abilities; 3/ increased emphasis on reflection; and 4/ increased professional status.

**Conclusions:**

A well-structured program based on the needs of preceptors and developed in partnership between educational and clinical settings seems to be successful in terms of preceptors’ perceived increase of their competence, abilities and professional status. What seems to be missing, not only from the current study but also from previous research, is to what extent properly prepared preceptors impact on student learning and this needs to be further investigated.

## Background

For health care professionals, clinical practice is a vital part of the education, and in several countries teaching is a regulated part of the role of nurses and health care staff [1–3]. Clinical practice involves preceptorship where experienced clinicians teach, reflect and assess the performance of students [4]. Carlson [5] described how precepting takes place in a learning environment where the preceptor strives to create a safe and meaningful interactive relationship with the student. During this process, preceptors need to promote reflection as an educational strategy to enhance critical thinking and problem-solving skills, and thereby, support the student’s ability to implement generalized theoretical knowledge into patient-centred problems within each specific health-care context. Preceptorship also entails for clinicians to create a supportive learning and working climate where students or newcomers are given opportunities to develop professional competence. However; several previous studies report the challenges connected to the preceptor role, for example, precepting the unsafe student [4], lack of allocated time for precepting [2], and lack of proper education in pedagogics [6, 7]. The added responsibility of taking on the teaching of students during clinical practice; thus, balancing clinical and educational demands, might lead to feelings of stress and burn-out [8], and dissatisfaction with the preceptor role [9]. Being a skilled and experienced professional, a characteristic highly valued by students [10], is not automatically linked to being a skilled educator as teaching of a subject is a completely different story. Therefore, it is not surprising that a number of studies argue for the development of further pedagogical education for health care professionals who are precepting students [1, 5, 11] focusing: reflection, critical thinking, communication and adult learning principles [12]. Carlson et al. [13] in their study on preceptors’ teaching strategies during clinical practice did not differentiate between preceptors with or without formal pedagogical education; however, the findings indicated that preceptors who had undergone preceptor training emphasised the importance of reflective questioning. This is further substantiated in a study by Chang et al. [14] concluding that receiving training in communication skills was deemed as the most important aspect with regards to clinical usefulness as a preceptor. It has also been reported that preceptors who had participated in educational initiatives were better prepared to address challenges and were more satisfied with the preceptor role [9], and experienced increased self-efficacy in the preceptor role [15]. In conclusion, the literature seems to underline the importance of preceptor education [10, 16, 17]. Even though, the course content seems to share some commonalities, for example, adult learning principles, communication skills and feedback strategies, the educational programmes seem to differ considerably in duration; from two hour courses [18], one to two days’ workshops [3, 10, 19] to a two week long course [20], and few courses are presented as continuous professional development meriting academic credits. The aim of the current study was to evaluate preceptors’ experiences of preceptorship in clinical practice after completion of a credit bearing continuous professional development (CPD) course on advanced level.

## Methods

This is a small-scale interpretative qualitative study drawing data from focus group interviews and written accounts from reflective journals. Focus group interviews were considered suitable for exploring group practices, interactions, and capturing perceptions of preceptorship in clinical practice [21]. The participants were preceptors who had completed a CPD course on advanced level.

### The CPD course –Preceptorship in Clinical Practice

During the spring of 2012, a multi-disciplinary CPD course at advanced level (second cycle) was developed and implemented at a university in southern Sweden. The content of the course curriculum was guided, in part, on findings from a study that invited preceptors from the health care sector in southern Sweden (unpublished data by Bengtsson and Carlson, 2015) to express their expectations of such an initiative. Further, a panel of stakeholders from affiliated hospitals and lecturers from the university met at two occasions to discuss relevant subject areas that needed to be covered to meet the needs of preceptors taking part in the course. Based on the input from the different groups the CPD course was designed to assist preceptors to deepen their knowledge of preceptorship concerning planning, leading and implementing educational activities directed to students, new graduates and co-workers. The intended learning outcomes were described as:

- critically examine and apply various adult learning principles and educational theories in clinical practice
- implement and evaluate models for communication and interaction in preceptor-preceptee encounters
- independently plan and implement teaching assignments with regard to adequate educational methods, taken into account evidence-based, social and ethical aspects of preceptorship.

The CPD course carries a credit of 7.5 ECTS credits and is campus-based. In Sweden 1.5 ECTS credits equal 40 h of student activity including lectures, seminars, laboratory and self-directed studies. The course contains adult learning theories, communication techniques, educational models and ethical aspects of preceptorship. Learning activities in the CPD course vary from lectures to workshops, case studies and field studies where participants observe each other as critical friends providing structured feedback on teaching activities. Critical friends are colleagues with comparable educational background evaluating the work of each other by asking provocative questions; thus, offering critique of a person’s work as a friend [22, 23]. Individual learning outcomes and reflections on the learning activities are documented by each student in a personal e-portfolio administrated via the web-based learning platform. Green et al. [24] presented e-portfolios as means for students to record skills, experiences and achievements during a course. Participants are formally evaluated and graded by a written assignment at the end of the course.

### Sampling and participants

All participants who attended and completed the CPD course during spring 2012 (*n* = 27) were invited by e-mail to participate. The invitation included an information letter briefly describing the study aim and design, the interview process, asking consent to use part of the personal e-portfolio, ethical considerations and contact details to the first author (EC). Eight previous course attendants volunteered to take part in the focus group interviews. The participants had previous experiences as preceptors ranging from 5–20 years, had worked within their respective professions for 7–25 years, and their mean age was 45 years (26–63). Two focus groups with 4 participants in each group were established. The first group included two registered nurses (RNs), one occupational therapist (OT) and one biomedical scientist (BS) and the second group comprised three RNs and one OT. Of the eight participants one was male. Moreover, none of the 27 course participants opposed to their reflective journals being used; thereby, written accounts focusing perceived learning outcomes after completion of the course are part of the data.

### Data collection

The interviews were held six months after completion of the CPD course as it was considered important that participants would have had an opportunity to practice knowledge and skills gained during the course. Interviews were held at the university were the authors work, conducted by using a digital voice-recorder, and lasted 65 and 59 min respectively. The opening question initiating the discussions was phrased as: *Looking back on what you learned during the course, what do you consider most valuable for you as a preceptor?* The discussions ran freely as participants shared their stories, the researchers asked follow-up question when parts of the stories needed to be clarified. Typically these were phrased as: *Can you please explain what you mean or can you give an example?* The part of the e-portfolio that has been used for data collection was a reflection on how they perceived they had fulfilled their individual learning outcomes. These entries in the e-portfolio were written one week after completion of the course and after students were evaluated and graded. The written reflections were limited to a maximum of 500 words. The total amount of the 27 journals were 6965 words ranging between 127 and 542 (mean = 258) words, providing rich descriptions of perceived learning outcomes.

### Data analysis

Each interview was transcribed verbatim and read through several times by the first author (EC) to grasp an initial understanding of the discussions. Reliability was assured by cross-checking the recorded interviews against the transcriptions. Text from the written reflective journals was processed in a similar way by both authors reading through the text at several occasions and continuously reflecting on emerging meanings with respect to perceptions of preceptorship. All text has thereafter been analysed as one unit of analysis. Data were analysed through the process of naturalistic inquiry [25], and started by the authors, independently, identifying meaning units illustrating patterns describing how participants perceived preceptorship after completing the CPD course. The initial meaning units were reflected on and discussed by the authors to confirm the congruence to the original text, thereafter; sorted into subcategories. The meaning of each subcategory was explained and clarified (Table 1) to allow the emergence of the main category. Selected quotes and excerpts are used to represent participants’ thoughts on preceptorship, and were discussed until agreement between the authors to ensure credibility.

**Table 1:** Examples from the analysis process explaining the main category Leaders of Educational Development

Meaning unit	Sub category	Explain and clarify
“They [the colleagues] turn to me more frequently with all kinds of questions when it comes to teaching and that feels really good” (P3; FG 1)^a^	increased ability to give collegial support	Descriptions of how participants experience that colleagues turn to them for advice and support.
“My belief in myself, my self-efficacy if you want, has definitely improved. I know where to look for guidance and I am more inspired to do a good job. (P5; FG2).	increased trust in one’s abilities	Descriptions of how participants explain how they have gained inner strength through the acquired knowledge.
“I believe that reflection and why we do it has become much clearer to me, from now on I will set aside time for short daily reflections and once a week a more structured session for an hour or so” (Reflective journal #17).	increased emphasis on reflection	Descriptions of how participants have realised the importance of reflection and how they use reflection as an educational strategy.
“I really enjoy being a preceptor now, it’s like I am treated slightly different. I am not only a nurse, but a precepting nurse with knowledge and skill” (P8; FG2).	increased professional status	Descriptions of how they perceive that the new knowledge has contributed to that the complexity of preceptorship is acknowledged by others.

^a^P indicates participant and F G stands for Focus Group i.e., P 3, F G 1 = participant number 3 in focus group number 1

### Ethical considerations

The study followed the principals according to the Helsinki Declaration [26]. The Ethical Advisory Board at the Faculty Health and Society, Malmö University approved of the study (HS 60–11/919:2). Prior to the interviews the first author informed the participants verbally with regards to the issue of confidentiality, the right to withdraw from the study at any time without further explanation, and repeated the study aim. Informed consent was obtained, and no participant terminated participation in the focus group interviews. The first author (EC) has been involved in teaching and grading the students, and this might pose a risk that students felt obliged to participate. However; the written accounts in the reflective journals were completed by the students one week after they had finished the course and the interviews six months later. Thereby, were the participants no longer dependant on the first author in any respect. The second author (MB) has not been involved in teaching or grading the students.

### Findings

Our findings show that the participants who took part in and completed the CPD course had developed skills and competences they believed to be necessary to drive pedagogical development at their respective work-places. We illustrate this by the main category *Leading educational development* which is explained by the following sub-categories: 1/ *increased ability to give collegial support; 2/ increased trust in one’s abilities; 3/ increased emphasis on reflection; and 4/ increased professional status.* The participants explained, in interviews and reflective journals, how they had gained self-confidence in relation to the preceptor role through the different learning activities. It was also described that the new knowledge and skills would be put to use to enable collegial discussions on precepting and clinical learning, as well as to implement new educational models for students.

“I have gained so much confidence, and I know that I have the ability to implement new ideas on how we precept our students; even though, it might be quite challenging” (Reflective journal # 15).

### Increased ability to give collegial support

After participating in the CPD course the participants related how they experienced that their colleagues turned to them to a larger extent, asking for educational advice. This reinforced a feeling of competence with regards to the preceptor role.

“They [the colleagues] turn to me more frequently with all kinds of questions when it comes to teaching and that feels really good” (P 3, Focus group 1).

However, this was not only felt in relation to increased knowledge of how to precept students, but it was also explained, that the acquired knowledge could be used to support less experienced preceptors.

“I will continue to reinforce the importance of preceptorship, if I could introduce my colleagues to the idea of supporting each other as critical friends that would really be something” (P 2, Focus group 1).

The idea of implementing a support system of critical friends was also elaborated on in the reflective journals “a critical friend might be one way forward if we want to strengthen the preceptor role” (Reflective journal #5), and most preceptors agreed that implementing new ideas would from now on be their responsibility.

### Increased trust in one’s abilities

Reflective journals as well as discussions in the focus groups disclosed how participants experienced an increased trust in their abilities as preceptors. This was described as having gained inner strength and the courage to try new approaches to precepting. On the one hand, being more creative and use a variety of pedagogical strategies were discussed among participants as one example of how they trusted their abilities; on the other hand, this was also explained in terms of being more structured as their organizational skills had improved. Participants expressed how they thought differently about the organization of students’ clinical experiences, and how students’ learning activities ought to be structured to support students’ achieving their learning outcomes.

“My belief in myself, my self-efficacy if you want, has definitely improved. I know where to look for guidance and I am more inspired to do a good job” (P 5, Focus group 2).

Participants also talked about how they used to feel uncomfortable and unsure when they had to fail an unsafe or under-achieving student. After participating in the course they expressed how the new knowledge and the improved communication skills helped them to be better prepared and courageous in situations they perceived as difficult.

“I am not afraid to tell the student when something is not just good enough or that I might have to fail the student…I have some inner strength now, and it makes it so much easier” (P 7, Focus group 2).

“I know that I have the necessary knowledge and it is no big deal any more if I need to discuss more sensitive matters with my student”(Reflective journal # 8).

### Increased emphasis on reflection

Most participants discussed the importance of critical thinking skills and problem-solving abilities for health care professionals. They agreed that facilitating the development of these abilities of their students was one major responsibility as preceptors.

“I believe that reflection and why we do it has become much clearer to me, from now on I will set aside time for short daily reflections and once a week a more structured session for an hour or so” (Reflective journal # 17).

The preceptors perceived that they had gained more understanding of how they could use reflection as an educational tool working with the students. They explained that their didactical approach had shifted from teacher-oriented to learner-oriented; thus, placing increased student activity in focus.

“It used to be a lot about me teaching, lecturing, talking, but I have realised how effective it can be if I listen more to the student, take a step back, and just ask more questions” (P 6, Focus group 2).

This was also illustrated by several entries in the reflective journals where participants wrote about their ambition to use more reflective questioning, and listen to their students without being judgmental or drawing hasty conclusions.

“I think that what I have learnt the most is that I need to listen more, not freely handing out all the answers to my students, but letting them present their ideas and suggestions, that is being reflective” (Reflective journal #9).

### Increased professional status

One of the perceived benefits of having participated in the CPD course was a feeling of increased professional status. Participants talked about how they had experienced that their colleagues turned to them for advice, and that preceptorship was discussed in collegial groups as a complex and advanced role that you needed to be properly prepared for. They concluded that the changes in attitudes were due to them taking part in a course on advanced level, thus giving legitimacy to the preceptor role.

“I really enjoy being a preceptor now; it’s like I am treated slightly different. I am not only a nurse, but a precepting nurse with knowledge and skills” (P 8, Focus group 2).

Participants also expressed the view that, not only did the CPD course increase the personal professional status, but also the overall status of the work-place.

“If the word goes around that this ward has a lot of well-prepared preceptors it might be easier to recruit and keep new grads” (Reflective journal #3).

Participants commented on the benefits for the work-place being associated with properly educated preceptors and emphasised the profound impact preceptorship can have on students’ perceptions of the clinical learning experience, which in the end will impact on recruitment of new staff.

## Discussion

This study provides some promising evidence for the contribution of a professional development course for preceptors with regards to increased confidence as leaders of educational development. The reported benefits from participating in the CPD course, that is, increased ability to give collegial support; increased trust in one’s abilities; increased emphasis on reflection; and increased professional status may be explained by a course targeting the needs of preceptors as its development was guided by input not only from preceptors but also service providers. This confirms the findings from a study by Jeggels et al. [20] who concluded that collaborative partnerships are imperative to the success of preceptorship education. Hilli et al. [6] argue that a supportive environment is fundamental for both preceptors and students. Based on our findings, where preceptors expressed that it is beneficial for a work-place in terms of recruiting new graduates to be known as a facilitating learning environment with educated preceptors, we will accentuate that a successful partnership starts already during the planning phase of a new course.

Even though, we did not set out to measure self-efficacy, the findings from the current study can be supported by Bandura’s theory of self-efficacy [27, 28]. Preceptors disclosed a sense of increased competence with regards to, for example, providing collegial support and handling the unsafe or under-achieving students. This finding is particularly noteworthy as McCarthy and Murphy [29] found that failing a student was experienced as difficult by preceptors and therefore underreported. This is not to say that preceptors in our study found it any less difficult, rather they had learnt how to use the new knowledge and the improved communication skills. Further, Larsen and Zahner [30] pointed to how self-efficacy is reliant on vicarious experiences and Bandura [27] explained how the observation of others can guide new behaviours and actions. Preceptors in our study had shadowed each other as critical friends during field studies and this experience was perceived as a support system for experienced as well as less experienced preceptors. This supports findings by Smedley et al. [15] who recommended that preceptor programs should include communication skills, approaches to learning, feedback and reflection, all skills that are practiced during shadowing as in our study. However, Lauder et al. [28] point to that self-efficacy is not the only answer to competence-linked change. Thereby; further research specifically addressing the experiences of critical friends as an educational activity in preceptor programs are recommended as such studies would contribute to the data on preceptor competence and self-efficacy. Moreover, participants in the current study expressed an increased understanding of reflection as an educational strategy; thereby, confirming findings from several previous studies [13, 19, 31]. This can, on the one hand, be explained by increased theoretical knowledge facilitated by lectures and seminars. On the other hand, the use of the e-portfolios where participants recorded and reflected on their learning [24] probably encouraged self-reflection, visualized the learning process and provided evidence of competence development; however, whether e-portfolios is a viable tool for recording and evaluating preceptor competence needs to be further investigated.

Feelings of stress, dissatisfaction with the preceptor role, and work overload for preceptors have been frequently reported in the literature [3, 8, 9, 30]. This is to a certain extent refuted in the current study where preceptors experienced a sense of increased professional status after the completion of the CPD course. This is a unique feature of the current study, and we are not aware of any previous studies discussing preceptorship in terms of professional status. We would propose that increased professional status, on the one hand, can be explained by a sense of intrinsic rewards of preceptorship previously reported as personal and professional development [32], updated clinical and scientific knowledge and job satisfaction [33]; on the other hand, we will argue that increased professional status is a consequence of having participated in a credit bearing course on advanced level where preceptors were formally evaluated and graded in an academic setting. Although, the course presented in the current study was campus-based with preceptors attending lectures and seminars twice a month it was not mentioned as a hindrance contrasting the findings of Luhanga et al. [16] who reported scheduling challenges and heavy work load as major barriers to participation. This can probably be accredited to the previously discussed partnership among all stakeholders collaborating in the development of the CPD course. This approach to course development gives legitimacy to participation in the course and to the activities and efforts required to achieve the learning outcomes.

### Limitations

This is a small single site study and therefore, no general conclusions can be drawn. However; even though data were collected at two different times and with different methods for data collection the experiences narrated and described by participants were corroborated across methods ensuring trustworthiness of the findings. Neither were any differences between health care professions detected during analysis of data, thus adding to trustworthiness. The non-hierarchical comparisons of written text and transcribed text from the interviews disclosed that the data were cohesive contributing to a richer understanding of the experiences of participating preceptors. There is a risk of response bias as we applied a purposeful approach to sampling for the focus group interviews which might have resulted in that only the most interested in precepting participated; thereby, limiting the generalisability.

## Conclusions

A well-structured program based on the needs of preceptors and developed in partnership between educational and clinical settings seems to be successful in terms of preceptors’ perceived increase of their competence, abilities and professional status. With the growing demand for clinical placements and the dependence of health care education on clinical preceptors it is vital that preceptor programs address the challenges preceptors meet. However, what seems to be missing, not only from the current study but also from previous research is to what extent educated and properly prepared preceptors impact on student learning and this needs to be further investigated.

## Abbreviations

MB: Mariette Bengtsson
EC: Elisabeth Carlson
CPD: Continuous Professional Development

## References

[1] Panzavecchia L, Pearce R (2014). Are preceptors adequately prepared for their role in supporting newly graduated staff?. Nurs Educ Today.

[2] Carlson E, Pilhammar E, Wann-Hansson C (2010). Time to precept: Supportive and Limiting Conditions for Precepting Nurses. J Adv Nurs.

[3] Ford K, Courtney-Pratt H, Fitzgerald M (2013). The development and evaluation of a preceptorship program using a practice development. Aust J Adv Nurs.

[4] Earle-Foley V, Myrick F, Luhanga F, Yonge O (2012). Preceptorship: Using an Ethical Lens to Reflect on the Unsafe Student. J Prof Nurs.

[5] Carlson E (2013). Precepting and Symbolic Interactionism: A Theoretical Look at Nursing Practice. J Adv Nurs.

[6] Hilli Y, Melender H-L, Salmu M, Jonsén E (2014). Being a preceptor- A Nordic qualitative study. Nurs Educ Today.

[7] Hyrkäs K, Shoemaker M (2007). Changes in the preceptor role: re-visiting preceptors’perceptions of benefits, rewards, support and commitment to the role. J Adv Nurs.

[8] Bourbonnais FF, Kerr E (2007). Preceptoring a student in the final clinical placement: Reflections from nurses in a Canadian hospital. J Clin Nurs.

[9] O’Brien A, Giles M, Dempsey S, Lynne S, McGregor M, Kable A, Parmenter G, Parker V (2014). Evaluating the preceptor role for pre-registration nursing and midwifery student clinical education. Nurs Educ Today.

[10] Heffernan C, Heffernan E, Brosnan M, Brown G (2009). Evaluating a preceptorship programme in South West Ireland: perceptions of preceptors and undergraduate students. J Nurs Manag.

[11] Öhman A, Hägg K, Dahlgren L (2005). A stimulating, practice-based job facing increased stress-/ Clinical supervisors’ perceptions of professional role, physiotherapy education and the status of the profession. Adv Physiother.

[12] Baltimore JJ (2004). The Hospital Clinical Preceptor: Essential Preparation for Success. J Contin Educ Nurs.

[13] Carlson E, Wann-Hansson C, Pilhammar E (2009). Teaching during clinical practice: Strategies and techniques used by preceptors in nursing education. Nurs Educ Today.

[14] Chang C-C, Lin L-M, Chen I-H, Kang C-M, Chang W-Y (2015). Perceptions and experiences of nurse preceptors regarding their training courses: A mixed method study. Nurs Educ Today.

[15] Smedley A, Morey P, Race P (2010). Enhancing the Knowledge, Attitudes and Skills of Preceptors: An Australian Perspective. J Contin Educ Nurs.

[16] Luhanga LF, Dickieson P, Mossey SD (2010). Preceptor Preparation: An Investment in the Future Generation of Nurses. Int J Nurs Educ Scholarsh.

[17] Mårtensson G, Engström M, Mamhidir A-G, Kristofferzon M-L (2013). What are the structural conditions of importance to preceptors’ performance?. Nurs Educ Today.

[18] Sandau KE, Halm M (2011). Effect of a Preceptor Education Workshop: Part 2. Qualitative Results of a Hospital-Wide Study. J Contin Educ Nurs.

[19] Henderson A, Fox R, Malko-Nyhan K (2006). An evaluation of Preceptors’ Perceptions of Educational Preparation and Organizational Support for Their Role. J Contin Educ Nurs.

[20] Jeggels JD, Traut A, Africa F (2013). A report on the development and implementation of a preceptorship training programme for registered nurses. Curationis.

[21] Creswell JW (2013). Qualitative Inquiry & Research Design.

[22] Costas AL, Kallick B (1993). Through the lens of a critical friend. Educ Leadersh.

[23] Dahlgren L-O, Eriksson BE, Gyllenhammar H, Korkeila M, Sääf-Rothoff A, Wernersson A, Seeberger A (2006). To be and to have a critical friend in medical teaching. Med Educ.

[24] Green J, Wyllie A, Jackson D (2014). Electronic portfolio in nursing education: A review of the literature. Nurs Educ Pract.

[25] Lincoln YS, Guba EG (1985). Naturalistic Inquiry.

[26] WMA www.wma.net/en/30publications/10policies/b3/index.html. Accessed March 10^th^, 2015.

[27] Bandura A (1977). Self-efficacy: Toward a unifying theory of behavioural change. Psychol Rev.

[28] Lauder W, Holland K, Roxburgh M, Topping K, Watson R, Johnson M (2008). Measuring competence, self-reported competence and self-efficacy in pre-registration students. Nurs Stand.

[29] McCarthy B, Murphy S (2010). Preceptors’ experiences of clinically educating and assessing undergraduate nursing students: and Irish context. J Nurs Manag.

[30] Larsen R, Zahner SJ (2011). The impact of Web-delivered Education on Preceptor Role Self-Efficacy and Knowledge in Public Health Nurses. Public Health Nurs.

[31] Myrick F, Luhanga F, Billay D, Foley V, Yonge O (2012). Putting the Evidence into Preceptor Preparation. Nurs ResPract.

[32] Muir J, Ooms A, Tapping J, Marks-Maran D, Phillips S, Burke L (2013). Preceptors’ perceptions of a preceptorship programme for newly qualified nurses. Nurs Educ Today.

[33] Natan BM, Qeadan H, Egbaria W (2014). The commitment of Israeli nursing preceptors to the role of preceptor. Nurs Educ Today.

